# Numerical Investigation of the Impact of Processing Conditions on Burr Formation in Carbon Fiber-Reinforced Plastic (CFRP) Drilling with Multiscale Modeling

**DOI:** 10.3390/ma18061244

**Published:** 2025-03-11

**Authors:** Guangjian Bi, Xiaonan Wang, Yongjun Shi, Cheng Zhang, Xuejin Zhao

**Affiliations:** 1 College of Mechanical and Electronic Engineering, Shandong University of Science and Technology, Qingdao 266590, China; 2 College of Mechanical and Electrical Engineering, China University of Petroleum (East China), Qingdao 266580, China

**Keywords:** CFRPs, drilling, burr distribution, multiscale simulation, constitutive model, tool geometry, processing parameter

## Abstract

Burrs generated during the drilling of carbon fiber-reinforced plastics (CFRPs) would seriously reduce the service life of the components, potentially leading to assembly errors and part rejection. To solve this issue, this paper proposed a finite element (FE) model with multiscale modeling to investigate the formation and distribution of burrs at various processing conditions. The FE model comprised the microscopic fiber and resin phases to predict the formation process of burrs, while part of the CFRP layers was defined to be macroscopic equivalent homogeneous material (EHM) to improve the computational efficiency. A progressive damage constitutive model was proposed to simulate the different failure modes and damage propagation of fibers. The impact of strain rate on the mechanical properties of the resin and CFRP layers was considered during the formulation of their constitutive models. With this numerical model, the formation process of the burrs and the drilling thrust force were accurately predicted compared to the experimental measurements. Then, the burr distributions were analyzed, and the influences of the drill bit structures and drilling parameters on burrs were assessed. It was concluded that the burrs were easily generated in the zones with 0° to 90° fiber cutting angles at the drilling exit. The sawtooth structure could exert an upward cutting effect on burrs during the downward feed of the tool; thus, it is helpful for the inhibition of burrs. More burrs were produced with higher feed rates and reduced spindle speeds.

## 1. Introduction

Due to the extraordinary mechanical properties of carbon fiber-reinforced plastics (CFRPs), including high specific strength, high specific modulus, and excellent damage tolerance, the applications of high-performance parts made of these composites have shown an obviously increasing trend in aerospace, transportation, and energy fields [1,2]. The CFRP parts are required to be joined with other components by bolts or rivets in the assembly process. Therefore, drilling is an essential process for CFRP component assembly [3,4]. However, CFRP drilling with high quality is still a considerable challenge. CFRP workpieces are commonly laminated by several prepregs with various fiber orientations, and each of the layers is composed of fibers and resins. These special material compositions and structural characteristics make the machining of CFRPs quite different from that of the traditional metals. Especially, the interlaminar bond strength of the CFRPs is weak, and the fibers with high strength are difficult to cut while the resin is easy to remove. In this situation, serious damages, including fiber pull-out, delamination, and burrs are generated easily in the drilling of CFRPs [5,6]. These damages would significantly reduce the bearing capacity of the CFRPs, and shorten the service life of the high-end equipment. Even worse, some damages could lead to serious assembly errors and part rejection [7,8]. Consequently, a primary objective for composite researchers is to investigate the mechanisms underlying the formation and distribution of drilling-induced damages in CFRPs, as well as to assess the impact of drill bit geometries and drilling parameters on such damages.

Generally, the damages in CFRP drilling could be observed and measured directly with the experimental method. In the existing investigations, advanced equipment, including high-speed cameras, was adopted to observe the damages at the drilling exit of CFRPs, and the material fracture and damage propagation at the exit were analyzed [9,10]. The influences of tool geometry, spindle speed, and feed rate on the drilling quality were experimentally studied by using drill bits such as one-shot drill and twist drill [11,12]. Moreover, based on parameter analysis and multiple linear regression, empirical formulas were reported to predict delamination factors under different processing conditions [13,14]. However, due to the high cutting speed in drilling, it is hard to clearly observe the formation of the damages at hole exit based on existing equipment, and the measurement of the damages inside the CFRP workpiece is more difficult. When studying the effect of processing conditions on drilling damage, a large number of experiments are often required, which would waste a high volume of composite materials. Furthermore, the empirical formula used to predict the damages is only effective within a certain range of parameters.

By contrast, the finite element (FE) simulation possesses the ability to model the continuous dynamic drilling process of CFRPs in three-dimensional (3D) space, and the stress distribution and damage formation mechanism of materials could be conveniently obtained. The damage extent under different drilling conditions could be easily predicted by modifying the tool geometries and processing parameters of the numerical model. In addition, the FE simulation is a cost-saving method, which only consumes a small amount of CFRP materials during the experiments for simulation verification. Therefore, numerical models for the CFRP drilling have been developed by researchers, and the influences of drill bit geometries and drilling parameters on thrust force and delamination have been explored. In the simulation proposed by Isbilir et al. [15,16], the intralaminar failure of CFRPs was determined based on Hashin criteria, and the interlayer interface was modeled with cohesive behavior. The model was used to predict the thrust force and delamination factors when drilling CFRPs by step drills with various stage ratios and twist drills. On the basis of cohesive elements, Feito et al. [17,18,19] developed a 3D simulation model for the drilling of multidirectional CFRPs with twist drill and step drill. With this model, the changes in the delamination and thrust force under different tool geometry and processing parameters were studied. It was found that the feed rate has a substantial impact on the thrust force and the occurrence of delamination. Liu et al. [8,20,21] investigated the drilling damage of CFRPs with the FE method, and the delamination factor and thrust force increased sharply with the rise of the feed rate. The results also show that the machining scheme with lower feed rate and higher spindle speed is conductive to improve the quality of holes.

**Figure 1:**
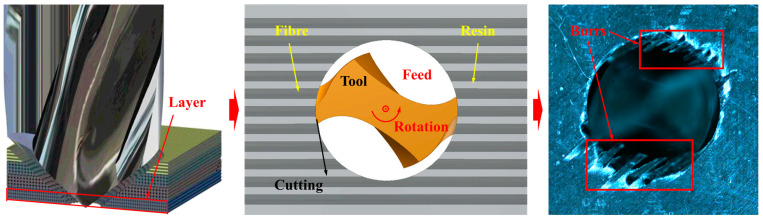
Schematic diagram of burrs.

It could be found that the majority of FE models established in recent studies for simulating CFRP drilling were built on the assumption of an equivalent homogeneous material (EHM), in which each layer of the composites was considered to be homogeneously equivalent. Moreover, these simulations were focused on the research of delamination. However, the burr is also a significant damage mode that occurs in CFRP drilling, yet it was seldom investigated numerically. Overlooking this type of damage in FE modeling would impede the accurate prediction of the mechanical behavior of drilled composites. The reason could be attributed to the fact that the burrs can accelerate the initiation and growth of delamination, as well as fiber pull-out due to the continuous scraping of the drill bit edges. Furthermore, although burrs can be removed through post-processing, this adds to the overall processing time. Consequently, to improve the drilling quality and efficiency, the formation mechanism of burrs, as well as its variations with the tool geometries and processing parameters, should be figured out. It is important to point out that the formation of burrs is largely dependent on the localized deformation and failure process of the fiber and resin phases on the micro scale (see Figure 1). Simulations based on the EHM assumption, which neglects the interaction of the drill bit with fibers and resins, are clearly unsuitable for studying burrs [22]. Under this circumstance, a numerical model that incorporates both fibers and resins should be developed to explore the mechanisms behind burr formation and to further reveal the impact of processing conditions on this type of damage.

In this paper, a three-dimensional (3D) FE model with multiscale modeling was developed to investigate the distributions of burr in the drilling of CFRPs under various processing conditions. The FE model comprised the microscopic fiber and resin phases to simulate the burr formation. Meanwhile, part of the CFRP layers in the numerical model was defined to be macroscopic EHM to reduce the computational time. The mechanical behaviors of all these material phases were characterized by defining specific constitutive models, failure criteria, and damage evolution laws. More notably, a progressive damage constitutive model was proposed to predict the different failure modes and damage evolution processes of fibers. The strain rate effect on the mechanical properties of the resin and CFRP layers was also considered during the formulation of their constitutive models. Based on this numerical model, the removal process of the fibers and resins at hole exit during the drilling of multidirectional CFRPs was simulated, and the thrust force was obtained. Then, experiments on CFRP drilling were carried out to verify the numerical model. Subsequently, the distribution laws of the burr were analyzed using the FE model, and the influences of the drill bit structures and drilling parameters on the burr factor were assessed.

## 2. Numerical Procedure

The multiscale numerical model used to predict the CFRP drilling was established using the Abaqus/CAE 2016 (Dassault Systèmes, Paris, France). Since the cutting speed during the processing is high and the contact between the drill bit and workpiece is complex, the numerical procedure was solved in the dynamic explicit analysis module. In this section, the geometrical model and material definitions of the FE model are detailed.

### 2.1. Geometrical Model

#### 2.1.1. Geometries of the Composite Workpiece and Drill Bit

**Figure 2:**
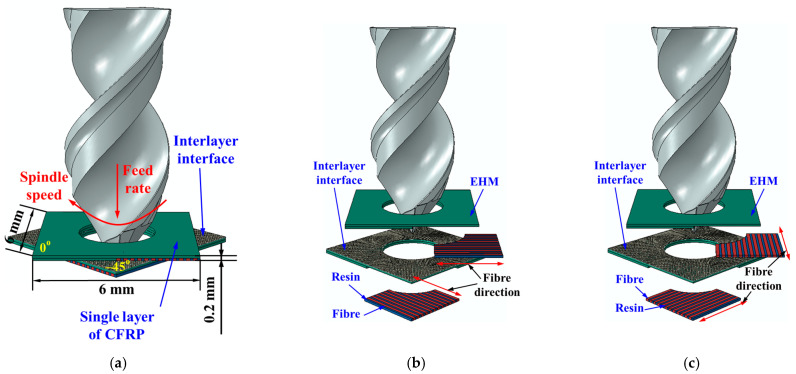
Geometrical model of the FE simulation model for CFRP drilling: (**a**) Details of the model. (**b**) Distributions of the constituent phases in Simulation-I. (**c**) Distributions of the constituent phases in Simulation-II.

Figure 2 illustrates the geometrical model of the multiscale numerical model. The workpiece was defined as a deformable solid, which consisted of carbon fibers, resins, interlayer interfaces, and CFRP layers that were treated as EHM. To reduce the computational time while ensuring precision, several strategies were adopted to optimize the geometric model of the CFRPs. Firstly, the workpiece was simplified to only two plies with a stacking sequence of 0/−45 (see Figure 2a). Secondly, considering that the diameter of the tool has less effect on the drilling-induced damage of CFRPs [23], the diameter of the hole supposed to be processed was determined to be 4 mm to reduce the size of the workpiece. Consequently, the dimension of each single layer in the workpiece was defined to be 6 mm × 6 mm × 0.2 mm. Thirdly, a 3 mm diameter pre-drilled hole was defined on the workpiece to minimize the number of integration points included in the calculation. Fourth, the cutting action on fibers under 0° to 180° cutting angles during the drilling was divided into two simulation processes named Simulation-I (see Figure 2b) and Simulation-II (see Figure 2c) for parallel computation. To be specific, Simulation-I was utilized to predict the cutting of fibers under 0° to 90° cutting angle, while the fiber cutting angles in Simulation-II covered from 90° to 180°. Fifth, a multiscale modeling was adopted during the simulation. As shown in Figure 2, only a small area in each CFRP layer was defined to be a fiber–resin combined zone, and the other part of the CFRP layers was assumed to be EHM. The fiber–resin combined zone was further divided into a fiber zone and a resin zone based on the fiber volume fraction. Moreover, zero-thickness cohesive elements were used to simulate the interlayer interface, which bonded the CFRP layers together. The material distributions and fiber orientations in Simulation-I and Simulation-II are shown by Figure 2b and Figure 2c, respectively.

The twist drill bit with a diameter of 4 mm was utilized in the FE model, and the tool geometries are listed in Table 1. The drill bit was modeled in Autodesk Inventor 2020 (Autodesk, San Francisco, CA, USA) and then imported into Abaqus/Explicit. The influence of tool wear on the burr distribution was not involved in the investigation; the drill bit was hence defined as an analytical rigid body.

With the aim of further enhancing the calculation efficiency of the numerical model, the materials in various zones of the workpiece were meshed with different sizes. More specifically, the elements in the fiber–resin combined zone were refined to simulate the actions of the drill bit on the fibers and resins accurately, and the element size was about 0.05 mm. While relatively coarse meshes with a dimension of about 0.1 mm were given in the EHM and interlayer interface areas to reduce the computational time [24]. The commonly used 8-node linear brick element with reduced integration (C3D8R) was applied in the fiber–resin combined zone and EHM zone, and they were generated by the sweep meshing technique. The enhanced hourglass control technique has been applied to suppress the hourglassing problem. The elements for the interlayer interface were set to be 8-node 3D cohesive elements (COH3D8), which were created by offsetting the elements in the EHM zone. In addition, 4-node linear tetrahedron elements without element deletion (C3D4) were defined for the drill bit.

#### 2.1.2. Boundary Conditions

The tie constraint method was employed to bind the fiber–resin combined zone, EHM, and interlayer interface together. For the nodes on the outer edge of the workpiece, all their degrees of freedom were restricted to prevent the CFRPs from moving. The movement of the drill bit in its radial direction was limited, and the rotational and translational velocities along the axial direction were set to the drill body, representing the spindle speed and feeding, respectively. The drilling parameters adopted commonly used values [4,25], as listed in Table 1.

Additionally, a surface-to-surface contact algorithm was utilized to predict the cutting and friction effects of the tool on the workpiece [16,26], and the behaviors along the contact surface, both normal and tangential, were defined. Specifically, the normal behavior was described based on a hard contact model, and a Coulomb friction algorithm with a 0.3 friction coefficient was utilized for describing the tangential behavior [27]. Furthermore, to avoid penetrations between each part of the CFRPs during the processing, a general contact algorithm was set between them.

### 2.2. Progressive Damage Constitutive Models for the Constituent Phases

In this subsection, progressive damage constitutive models were defined for the carbon fiber, epoxy resin, EHM, and interlayer interface to characterize their mechanical behaviors. Since the drill bit was treated as an analytical rigid body, only its density and modulus were set in the FE model.

#### 2.2.1. Carbon Fiber

Carbon fibers were assumed to be a transversely isotropic elastic material, and the constitutive relation of them before failure was defined by:

(1) $$\left\{\begin{array}{c} \varepsilon_{f1}\\ \varepsilon_{f2}\\ \varepsilon_{f3}\\ \gamma_{f23}\\ \gamma_{f31}\\ \gamma_{f12} \end{array}\right\}=\left[\begin{array}{cccccc} 1/E_{f1} & -\nu_{f12}/E_{f1} & -\nu_{f12}/E_{f1} & 0 & 0 & 0\\ -\nu_{f12}/E_{f1} & 1/E_{f2} & -\nu_{f23}/E_{f2} & 0 & 0 & 0\\ -\nu_{f12}/E_{f1} & -\nu_{f23}/E_{f2} & 1/E_{f2} & 0 & 0 & 0\\ 0 & 0 & 0 & 2(1+\nu_{f23})/E_{f2} & 0 & 0\\ 0 & 0 & 0 & 0 & 1/G_{f12} & 0\\ 0 & 0 & 0 & 0 & 0 & 1/G_{f12} \end{array}\right]\left\{\begin{array}{c} \sigma_{f1}\\ \sigma_{f2}\\ \sigma_{f3}\\ \tau_{f23}\\ \tau_{f31}\\ \tau_{f12} \end{array}\right\}$$

where *ε*_fi_ and *γ*_fij_ are the strains, and *σ*_fi_ and *τ*_fij_ are the stresses. *E*_fi_ and *G*_fij_ denote the moduli, and *ν*_fij_ represents Poisson’s ratio. i, j = 1, 2, 3, the subscripts f1, f2, and f3, signify the axial direction and two perpendicular radial directions, respectively. Additionally, the subscripts f12, f31, and f23 denote the 1-2 plane, 3-1 plane, and 2-3 plane, respectively.

The mechanical properties of carbon fiber vary obviously under axial tension and compression, as well as radial tension and compression loads, leading to diverse failure modes in response to multi-directional loadings. Therefore, by referring to the Hashin criteria [28], this paper introduced 3D criteria encapsulating four failure modes to determine the failure of carbon fiber.

Tensile failure in the axial direction:

(2) $$\sigma_{f1}\geq 0\to F_{f1}^{T}={\left(\frac{\sigma_{f1}}{X_{f}^{T}}\right)}^{2}+{\left(\frac{\tau_{f12}}{S_{f12}}\right)}^{2}+{\left(\frac{\tau_{f13}}{S_{f13}}\right)}^{2}=1$$

Compressive failure in the axial direction:

(3) $$\sigma_{f1}<0\to F_{f1}^{C}={\left(\frac{\sigma_{f1}}{X_{f}^{C}}\right)}^{2}+{\left(\frac{\tau_{f12}}{S_{f12}}\right)}^{2}+{\left(\frac{\tau_{f13}}{S_{f13}}\right)}^{2}=1$$

Tensile failure in the radial direction:

(4) $$\left\{\begin{array}{c} \sigma_{f2}\geq 0\to F_{f2}^{T}={\left(\frac{\sigma_{f2}}{Y_{f}^{T}}\right)}^{2}+{\left(\frac{\tau_{f12}}{S_{f12}}\right)}^{2}+{\left(\frac{\tau_{f23}}{S_{f23}}\right)}^{2}=1\\ \sigma_{f3}\geq 0\to F_{f3}^{T}={\left(\frac{\sigma_{f3}}{Z_{f}^{T}}\right)}^{2}+{\left(\frac{\tau_{f13}}{S_{f13}}\right)}^{2}+{\left(\frac{\tau_{f23}}{S_{f23}}\right)}^{2}=1 \end{array}\right.$$

Compressive failure in the radial direction:

(5) $$\left\{\begin{array}{c} \sigma_{f2}<0\to F_{f2}^{C}={\left(\frac{\sigma_{f2}}{Y_{f}^{C}}\right)}^{2}+{\left(\frac{\tau_{f12}}{S_{f12}}\right)}^{2}+{\left(\frac{\tau_{f23}}{S_{f23}}\right)}^{2}=1\\ \sigma_{f3}<0\to F_{f3}^{C}={\left(\frac{\sigma_{f3}}{Z_{f}^{C}}\right)}^{2}+{\left(\frac{\tau_{f13}}{S_{f13}}\right)}^{2}+{\left(\frac{\tau_{f23}}{S_{f23}}\right)}^{2}=1 \end{array}\right.$$

where $F_{f1}$, $F_{f2}$, and $F_{f3}$ are failure indexes. $X_{f}$, $Y_{f}$, and $Z_{f}$ represent the fiber strengths in one axial direction and two perpendicular radial directions, respectively. *S* is the shear strength. The superscripts T and C indicate the tension and compression effects, respectively.

Based on the experimental investigations [29,30,31], the carbon fibers would not break immediately after failure. During the cutting of CFRPs, the carbon fibers typically undergo localized damage initially, then the damage propagates progressively until complete removal of the fibers. Additionally, Li et al. [32] have predicted the CFRP cutting by defining the damage evolution process of the carbon fiber. Therefore, this research employed the commonly applied linear damage evolution law to characterize the irreversible evolution of the damage within fibers [33,34]. To be specific, a damage factor *d* was defined to regulate the stiffness degradation of fibers following any mode of failure. *d* was determined by strain variables, including the strain at each increment *ε*, the failure onset strain *ε*^0^, and the ultimate failure strain *ε^f^*, as shown by Equation (6). For every increment following the initiation of damage, the relevant effective stress components were multiplied by (1–*d*).

(6) $$d=\frac{\varepsilon^{f}(\varepsilon -\varepsilon^{0})}{\varepsilon (\varepsilon^{f}-\varepsilon^{0})}$$

The damage factors for the failure modes of axial tensile ($d_{f1}^{T}$) and compressive ($d_{f1}^{C}$), as well as radial tensile ($d_{f2}^{T}$, $d_{f3}^{T}$) and compressive ($d_{f2}^{C}$, $d_{f3}^{C}$), are as follows [33,34]:

(7) $$\begin{array}{l} d_{f1}^{T}=\frac{\varepsilon_{f1}^{\mathrm{fT}}(\varepsilon_{f1}-\varepsilon_{f1}^{0T})}{\varepsilon_{f1}(\varepsilon_{f1}^{\mathrm{fT}}-\varepsilon_{f1}^{0T})},\;d_{f1}^{C}=\frac{\varepsilon_{f1}^{\mathrm{fC}}(\varepsilon_{f1}-\varepsilon_{f1}^{0C})}{\varepsilon_{f1}(\varepsilon_{f1}^{\mathrm{fC}}-\varepsilon_{f1}^{0C})}\\ d_{f2}^{T}=\frac{\varepsilon_{f2}^{\mathrm{fT}}(\varepsilon_{f2}-\varepsilon_{f2}^{0T})}{\varepsilon_{f2}(\varepsilon_{f2}^{\mathrm{fT}}-\varepsilon_{f2}^{0T})},\;d_{f2}^{C}=\frac{\varepsilon_{f2}^{\mathrm{fC}}(\varepsilon_{f2}-\varepsilon_{f2}^{0C})}{\varepsilon_{f2}(\varepsilon_{f2}^{\mathrm{fC}}-\varepsilon_{f2}^{0C})}\\ d_{f3}^{T}=\frac{\varepsilon_{f3}^{\mathrm{fT}}(\varepsilon_{f3}-\varepsilon_{f3}^{0T})}{\varepsilon_{f3}(\varepsilon_{f3}^{\mathrm{fT}}-\varepsilon_{f3}^{0T})},\;d_{f3}^{C}=\frac{\varepsilon_{f3}^{\mathrm{fC}}(\varepsilon_{f3}-\varepsilon_{f3}^{0C})}{\varepsilon_{f3}(\varepsilon_{f3}^{\mathrm{fC}}-\varepsilon_{f3}^{0C})} \end{array}$$

where the damage onset strains and the ultimate failure strains were given by:

(8) $$\begin{array}{l} \varepsilon_{f1}^{0T}=\frac{X_{f}^{T}}{E_{f1}},\;\varepsilon_{f2}^{0T}=\frac{Y_{f}^{T}}{E_{f2}},\;\varepsilon_{f3}^{0T}=\frac{Z_{f}^{T}}{E_{f3}}\\ \varepsilon_{f1}^{0C}=\frac{X_{f}^{C}}{E_{f1}},\;\varepsilon_{f2}^{0C}=\frac{Y_{f}^{C}}{E_{f2}},\;\varepsilon_{f3}^{0C}=\frac{Z_{f}^{C}}{E_{f3}} \end{array}$$

(9) $$\begin{array}{l} \varepsilon_{f1}^{\mathrm{fT}}=\frac{2G_{f1C}^{T}}{X_{f}^{T}L^{c}},\;\varepsilon_{f2}^{\mathrm{fT}}=\frac{2G_{f2C}^{T}}{Y_{f}^{T}L^{c}},\;\varepsilon_{f3}^{\mathrm{fT}}=\frac{2G_{f3C}^{T}}{Z_{f}^{T}L^{c}}\\ \varepsilon_{f1}^{\mathrm{fC}}=\frac{2G_{f1C}^{C}}{X_{f}^{C}L^{c}},\;\varepsilon_{f2}^{\mathrm{fC}}=\frac{2G_{f2C}^{C}}{Y_{f}^{C}L^{c}},\;\varepsilon_{f3}^{\mathrm{fC}}=\frac{2G_{f3C}^{C}}{Z_{f}^{C}L^{c}} \end{array}$$

Here, $G_{f1C}^{T}$, $G_{f1C}^{C}$, $G_{f2C}^{T}$, $G_{f2C}^{C}$, $G_{f3C}^{T}$, and $G_{f3C}^{C}$ denote the fracture toughness of fiber. *L^c^* represents the characteristic length of the meshed elements.

A user-defined subroutine (VUMAT) was developed to integrate this material model into Abaqus/Explicit. In the VUMAT, a state variable (SDV16) was introduced to manage element deletion resulting from the ultimate failure of fibers. To avoid excessive element distortion caused by minor localized material stiffness, which could potentially terminate the calculation, SDV16 was activated when *d* reached 0.99. This ensured that some residual stiffness remained upon element removal. The mechanical properties of the fiber were obtained by consulting manufacturers and referring to References [35,36], as summarized in Table 2.

#### 2.2.2. Epoxy Resin

The epoxy resin could be regarded as an isotropic elastoplastic material. The mechanical behavior of the resin prior to damage onset was divided into the elastic phase and plastic phase, contingent upon whether the internal stress of the material reached the elastic limit *σ*_me_. During the elastic phase, the stress could be calculated as follows:

(10) $$\sigma_{m}=E_{m}\varepsilon_{m}$$

where $E_{m}$ and $\varepsilon_{m}$ are the elastic modulus and strain, respectively. After the resin entered the plastic phase, a plastic constitutive law with isotropic hardening was employed.

The shear failure criterion [37] that was frequently utilized in previous studies [38,39] was adopted to determine the damage initiation of resin:

(11) $$\omega_{S}=\int \frac{d{\bar{\varepsilon }}^{\mathrm{pl}}}{{\bar{\varepsilon }}_{0}^{\mathrm{pl}}(\theta_{S},{\dot{\bar{\varepsilon }}}^{\mathrm{pl}})}=1$$

where $\omega_{S}$ is the failure index, and ${\bar{\varepsilon }}^{\mathrm{pl}}$ and ${\dot{\bar{\varepsilon }}}^{\mathrm{pl}}$ are the plastic strain and plastic strain rate, respectively. $\theta_{S}=(q+k_{s}p)/\tau_{\max}$ denotes the shear stress ratio [37]. ${\bar{\varepsilon }}_{0}^{\mathrm{pl}}$ represents the plastic strain at damage initiation.

After damage occurred, the damage factor *d*_m_ was used to control the stiffness degradation:

(12) $${\dot{d}}_{m}=\frac{L^{c}{\dot{\bar{\varepsilon }}}^{\mathrm{pl}}}{{\bar{u}}_{f}^{\mathrm{pl}}}=\frac{{\dot{\bar{u}}}^{\mathrm{pl}}}{{\bar{u}}_{f}^{\mathrm{pl}}}$$

The equivalent plastic displacement at failure reads [37]:

(13) $${\bar{u}}_{f}^{\mathrm{pl}}=\frac{2G_{\mathrm{mC}}}{\sigma_{\mathrm{mb}}}$$

where *σ*_mb_ and *G*_mC_ are the ultimate strength and fracture toughness, respectively.

**Figure 3:**
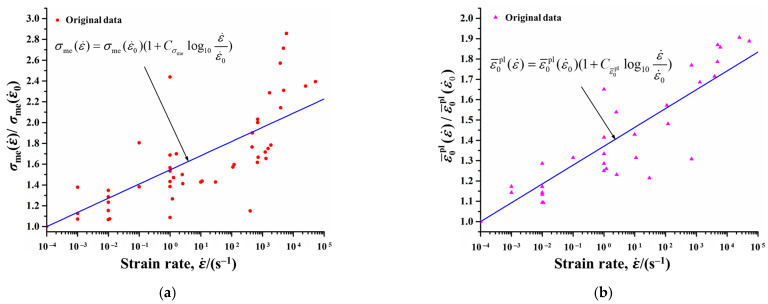
Variations in mechanical properties of resin with strain rate: (**a**) Elastic limit, *σ*_me_. (**b**) Plastic strain at damage initiation, ${\bar{\varepsilon }}_{0}^{\mathrm{pl}}$.

The above damage model could describe the mechanical response of resin under quasi-static loadings. However, the mechanical properties of resins vary substantially with the change in strain rate. Therefore, to ensure precise prediction of CFRP cutting, it is crucial to further incorporate the strain rate effect into the simulation. Nevertheless, in existing studies on CFRP cutting modeling, the variation in strain rate (e.g., ${\dot{\bar{\varepsilon }}}^{\mathrm{pl}}$ in Equation (11)) and its impact on the mechanical properties of resin were often ignored. To address this gap, the present work identified the mechanical properties (i.e., *σ*_me_, ${\bar{\varepsilon }}_{0}^{\mathrm{pl}}$) of resin across varying strain rates by referring to studies [40,41,42,43,44,45] first, and they were normalized to serve as the original data (represented as points in Figure 3). Then, the variation functions of *σ*_me_ and ${\bar{\varepsilon }}_{0}^{\mathrm{pl}}$ with respect to strain rate were fitted with a developed MATLAB 2017a (MathWorks, Natick, MA, USA) program and the original data, as shown by Equations (14) and (15) (see curves in Figure 3a and Figure 3b, respectively). Subsequently, these functions were implemented into the elastoplastic constitutive relationship and damage initiation criterion of the resin, respectively. In this manner, the mechanical response of resin at different strain rates during the CFRP cutting process was modeled.

(14) $$\sigma_{\mathrm{me}}(\dot{\varepsilon })=\sigma_{\mathrm{me}}({\dot{\varepsilon }}_{0})(1+C_{\sigma_{\mathrm{me}}}\log_{10}\frac{\dot{\varepsilon }}{{\dot{\varepsilon }}_{0}})$$

(15) $${\bar{\varepsilon }}_{0}^{\mathrm{pl}}(\dot{\varepsilon })={\bar{\varepsilon }}_{0}^{\mathrm{pl}}({\dot{\varepsilon }}_{0})(1+C_{{\bar{\varepsilon }}_{0}^{\mathrm{pl}}}\log_{10}\frac{\dot{\varepsilon }}{{\dot{\varepsilon }}_{0}})$$

In Equations (14) and (15), $\dot{\varepsilon }$ denotes the strain rate, and ${\dot{\varepsilon }}_{0}$ represents the reference strain rate. $\sigma_{\mathrm{me}}({\dot{\varepsilon }}_{0})$ and ${\bar{\varepsilon }}_{0}^{\mathrm{pl}}({\dot{\varepsilon }}_{0})$ are the *σ*_me_ and ${\bar{\varepsilon }}_{0}^{\mathrm{pl}}$ at reference strain rate, respectively. The material constants $C_{\sigma_{\mathrm{me}}}$ and $C_{{\bar{\varepsilon }}_{0}^{\mathrm{pl}}}$ are 0.1363 and 0.0927, respectively. Additionally, the mechanical properties of resin at the reference strain rate are listed in Table 3 [46].

#### 2.2.3. EHM in the CFRP Workpiece

The material model of the EHM was defined based on our previous research [47]. Specifically, it was assumed to have orthogonal anisotropic characteristics, and the elastic constitutive model formulated by Equation (16) was adopted to describe its material behavior before failure.

(16) $$\left[\begin{array}{l} \sigma_{1}\\ \sigma_{2}\\ \sigma_{3}\\ \tau_{23}\\ \tau_{31}\\ \tau_{12} \end{array}\right]=\left[\begin{array}{cccccc} C_{11} & C_{12} & C_{13} & 0 & 0 & 0\\ C_{12} & C_{22} & C_{23} & 0 & 0 & 0\\ C_{13} & C_{23} & C_{33} & 0 & 0 & 0\\ 0 & 0 & 0 & C_{44} & 0 & 0\\ 0 & 0 & 0 & 0 & C_{55} & 0\\ 0 & 0 & 0 & 0 & 0 & C_{66} \end{array}\right]\left[\begin{array}{l} \varepsilon_{1}\\ \varepsilon_{2}\\ \varepsilon_{3}\\ \gamma_{23}\\ \gamma_{31}\\ \gamma_{12} \end{array}\right]$$

In which,

(17) $$\begin{array}{l} C_{11}=E_{1}\left(1-\nu_{23}\nu_{32}\right)/\Delta \\ C_{12}=E_{2}\left(\nu_{12}+\nu_{32}\nu_{13}\right)/\Delta \\ C_{13}=E_{3}\left(\nu_{13}+\nu_{12}\nu_{23}\right)/\Delta \\ C_{23}=E_{3}\left(\nu_{23}+\nu_{21}\nu_{13}\right)/\Delta \\ C_{22}=E_{2}\left(1-\nu_{13}\nu_{31}\right)/\Delta \\ C_{33}=E_{3}\left(1-\nu_{12}\nu_{21}\right)/\Delta \\ C_{44}=G_{23}C_{55}=G_{31}C_{66}=G_{12}\\ \Delta =1-\nu_{12}\nu_{21}-\nu_{23}\nu_{32}-\nu_{13}\nu_{31}-2\nu_{21}\nu_{32}\nu_{13} \end{array}$$

Here, *σ*_i_ and *τ*_ij_ are the stresses, and *ε*_i_ and *γ*_ij_ are the strains. *E*_i_ and *G*_ij_ represent the moduli, and *ν*_ij_ is Poisson’s ratio. i, j = 1, 2, 3, which represent the longitudinal and transverse directions, as well as the through-thickness direction of the CFRP layer, respectively.

During the machining of CFRP laminates, different damage modes would initiate [48]. Therefore, this work utilizd Hashin–Puck combined criteria to predict the damage initiation of the EHM [28,49,50,51], and four distinct failure modes were defined individually.

Fiber tensile failure (*σ*_1_ ≥ 0) [28,49]:

(18) $$F_{f}^{T}={\left(\frac{\sigma_{1}}{X^{T}}\right)}^{2}+{\left(\frac{\tau_{12}}{S_{12}}\right)}^{2}+{\left(\frac{\tau_{13}}{S_{13}}\right)}^{2}=1$$

Fiber compressive failure (*σ*_1_ < 0) [28,49]:

(19) $$F_{f}^{C}={\left(\frac{\sigma_{1}}{X^{C}}\right)}^{2}+{\left(\frac{\tau_{12}}{S_{12}}\right)}^{2}+{\left(\frac{\tau_{13}}{S_{13}}\right)}^{2}=1$$

Matrix tensile failure (*σ*_2_ ≥ 0) [28,49]:

(20) $$F_{m}^{T}={\left(\frac{\sigma_{2}}{Y^{T}}\right)}^{2}+{\left(\frac{\tau_{12}}{S_{12}}\right)}^{2}+{\left(\frac{\tau_{23}}{S_{23}}\right)}^{2}=1$$

Matrix compressive failure (*σ*_2_ < 0) [50]:

(21) $$F_{m}^{C}={\left(\frac{\tau_{t}}{S_{23}^{A}-\mu_{t}\sigma_{n}}\right)}^{2}+{\left(\frac{\tau_{s}}{S_{12}-\mu_{s}\sigma_{n}}\right)}^{2}=1$$

where *F* denotes failure indexes. The subscripts f and m represent the fiber failure and matrix failure, respectively. *X*^T^ and *X*^C^ are the tensile strength and compressive strength of unidirectional CFRP laminates in the longitudinal direction, respectively. *Y*^T^ expresses the tensile strength in the transverse direction. *S*_ij_ denotes shear strengths. Based on the Puck theory [50,51], the unidirectional CFRPs fracture on a plane with the angle of *θ* in the through-thickness direction when subjected to compression loading in the transverse direction. $S_{23}^{A}$ is the shear strength perpendicular to the fiber direction on the fracture plane [52]:

(22) $$S_{23}^{A}=\frac{Y^{C}}{2\tan(\theta )}$$

where *Y*^C^ is the compressive strength in the transverse direction. *σ*_n_, *τ*_t_, and *τ*_s_ represent the stress components in normal, transverse, and longitudinal directions of the fracture plane, respectively:

(23) $$\begin{array}{l} \sigma_{n}=\sigma_{2}\cos^{2}\theta +\sigma_{3}\sin^{2}\theta +2\tau_{23}\sin\theta \cos\theta \\ \tau_{t}=\left(\sigma_{3}-\sigma_{2}\right)\sin\theta \cos\theta +\tau_{23}(\cos^{2}\theta -\sin^{2}\theta )\\ \tau_{s}=\tau_{31}\sin\theta +\tau_{21}\cos\theta \end{array}$$

where *μ*_t_ and *μ*_s_ are friction coefficients [52]:

(24) $$\mu_{t}=-\frac{1}{\tan(2\theta )},\;\mu_{s}=S_{12}\frac{\mu_{t}}{S_{23}^{A}}$$

Once the damage was initiated in the EHM, the linear damage evolution law was utilized to simulate the damage propagation, and the material stiffness was progressively reduced under the regulation of damage factor *d*. The damage factors for fiber tensile failure, fiber compressive failure, and matrix tensile failure are expressed as follows:

(25) $$d_{f}^{T}=\frac{\varepsilon_{1}^{\mathrm{fT}}(\varepsilon_{1}-\varepsilon_{1}^{0T})}{\varepsilon_{1}(\varepsilon_{1}^{\mathrm{fT}}-\varepsilon_{1}^{0T})},\;d_{f}^{C}=\frac{\varepsilon_{1}^{\mathrm{fC}}(\varepsilon_{1}-\varepsilon_{1}^{0C})}{\varepsilon_{1}(\varepsilon_{1}^{\mathrm{fC}}-\varepsilon_{1}^{0C})},\;d_{m}^{T}=\frac{\varepsilon_{2}^{\mathrm{fT}}(\varepsilon_{2}-\varepsilon_{2}^{0T})}{\varepsilon_{2}(\varepsilon_{2}^{\mathrm{fT}}-\varepsilon_{2}^{0T})}$$

where the damage onset strains and the ultimate failure strains are expressed as follows:

(26) $$\varepsilon_{1}^{0T}=\frac{X^{T}}{E_{1}},\;\varepsilon_{1}^{0C}=\frac{X^{C}}{E_{1}},\;\varepsilon_{2}^{0T}=\frac{Y^{T}}{E_{2}}$$

(27) $$\varepsilon_{1}^{\mathrm{fT}}=\frac{2G_{1C}^{T}}{X^{T}L^{c}},\;\varepsilon_{1}^{\mathrm{fC}}=\frac{2G_{1C}^{C}}{X^{C}L^{c}},\;\varepsilon_{2}^{\mathrm{fT}}=\frac{2G_{2C}^{T}}{Y^{T}L^{c}}$$

In which, $G_{1C}^{T}$, $G_{1C}^{C}$ and $G_{2C}^{T}$ are the fracture toughness. Regarding the compressive failure in the transverse direction, the damage factor was calculated by the strains on the fracture plane:

(28) $$d_{m}^{C}=\frac{\varepsilon_{\mathrm{mat}}^{\mathrm{fC}}(\varepsilon_{\mathrm{mat}}-\varepsilon_{\mathrm{mat}}^{0C})}{\varepsilon_{\mathrm{mat}}(\varepsilon_{\mathrm{mat}}^{\mathrm{fC}}-\varepsilon_{\mathrm{mat}}^{0C})}$$

where $\varepsilon_{\mathrm{mat}}=\sqrt{\langle \varepsilon_{n}^{2}\rangle +\gamma_{t}^{2}+\gamma_{s}^{2}}$. The strains on the fracture plane are expressed as follows:

(29) $$\begin{array}{l} \varepsilon_{n}=\varepsilon_{2}\cos^{2}\theta +\varepsilon_{3}\sin^{2}\theta +\gamma_{23}\sin\theta \cos\theta \\ \gamma_{t}=2\left(\varepsilon_{3}-\varepsilon_{2}\right)\sin\theta \cos\theta +\gamma_{23}(\cos^{2}\theta -\sin^{2}\theta )\\ \gamma_{s}=\gamma_{31}\sin\theta +\gamma_{21}\cos\theta \end{array}$$

The damage onset strain $\varepsilon_{\mathrm{mat}}^{0C}$ was acquired from $\varepsilon_{\mathrm{mat}}$ at the initiation of matrix compressive failure. The ultimate failure strain is as follows:

(30) $$\varepsilon_{\mathrm{mat}}^{\mathrm{fC}}=\frac{2G_{\mathrm{matC}}^{C}}{\sigma_{\mathrm{mat}}^{0C}L^{c}}$$

where $G_{\mathrm{matC}}^{C}$ is the fracture toughness. The damage onset stress $\sigma_{\mathrm{mat}}^{0C}$ is determined using the same method as the $\varepsilon_{\mathrm{mat}}^{0C}$:

(31) $$\sigma_{\mathrm{mat}}^{0C}=\sigma_{\mathrm{mat}}{|}_{F_{m}^{C}=1}=\sqrt{\langle \sigma_{n}^{2}\rangle +\tau_{t}^{2}+\tau_{s}^{2}}{|}_{F_{m}^{C}=1}$$

**Figure 4:**
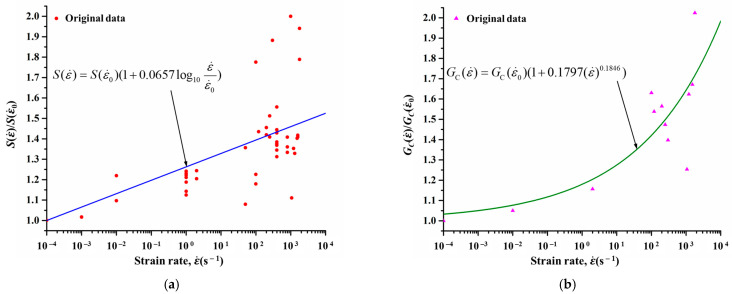
Changes in mechanical properties of the EHM with strain rate: (**a**) Strength. (**b**) Fracture toughness.

Since the mechanical properties of resin change significantly with the variation in strain rate, the material behavior of the EHM in the transverse direction is obviously affected by the strain rate. In this case, the relationship between the matrix-dominated mechanical properties of the CFRPs and the strain rate was further evaluated, and a strain-rate-dependent progressive damage constitutive model was developed for the EHM. Similar to the formulation of the material model of resin, the matrix-dominated strength and fracture toughness of the EHM at different strain rates were collected from previous studies [43,53,54,55,56,57,58,59,60,61] first, and they were normalized to serve as the original data (represented as points in Figure 4). Subsequently, the variation functions of the strength and the fracture toughness with respect to strain rate were fitted using a proposed MATLAB algorithm and the original data, which are shown by Equations (32) and (33) (see curves in Figure 4a and Figure 4b, respectively). Then, these functions were incorporated in the failure criteria and damage evolution rules of the EHM, respectively. Under this circumstance, the mechanical response of the EHM at different strain rates during the CFRP machining was modeled.

(32) $$S(\dot{\varepsilon })=S({\dot{\varepsilon }}_{0})(1+C_{S}\log_{10}\frac{\dot{\varepsilon }}{{\dot{\varepsilon }}_{0}})$$

(33) $$G_{C}(\dot{\varepsilon })=G_{C}({\dot{\varepsilon }}_{0})(1+C_{G_{C}}{\left(\dot{\varepsilon }\right)}^{N_{G_{C}}})$$

Here, *S* = *Y*^T^, *Y*^C^, *S*_12_, *S*_13_, *S*_23_; *G_C_* = $G_{2C}^{T}$, $G_{\mathrm{matC}}^{C}$. $S({\dot{\varepsilon }}_{0})$ and $G_{C}({\dot{\varepsilon }}_{0})$ are the strength and fracture toughness at reference strain rate, respectively. In addition, the material constants $C_{S}$, $C_{G_{C}}$, and $N_{G_{C}}$ are 0.0657, 0.1797, and 0.1846, respectively.

On the basis of Equations (32) and (33), the strain-rate-dependent damage model was formulated by modifying Equations (18)–(22), (24)–(28), and (30). The failure criteria involving the effect of strain rate are listed in Table 4. Here, $S_{23}^{A}(\dot{\varepsilon })$ and $\mu_{s}(\dot{\varepsilon })$ are expressed as follows:

(34) $$S_{23}^{A}(\dot{\varepsilon })=\frac{Y^{C}({\dot{\varepsilon }}_{2})}{2\tan(\theta )},\;\mu_{s}(\dot{\varepsilon })=S_{12}({\dot{\varepsilon }}_{12})\frac{\mu_{t}}{S_{23}^{A}(\dot{\varepsilon })}$$

Moreover, the damage evolution laws considering the strain rate effect could be expressed as follows:

(35) $$\begin{array}{l} d_{f}^{T}=\frac{\varepsilon_{1}^{\mathrm{fT}}(\varepsilon_{1}-\varepsilon_{1}^{0T})}{\varepsilon_{1}(\varepsilon_{1}^{\mathrm{fT}}-\varepsilon_{1}^{0T})},\;d_{f}^{C}=\frac{\varepsilon_{1}^{\mathrm{fC}}(\varepsilon_{1}-\varepsilon_{1}^{0C})}{\varepsilon_{1}(\varepsilon_{1}^{\mathrm{fC}}-\varepsilon_{1}^{0C})}\\ d_{m}^{T}(\dot{\varepsilon })=\frac{\varepsilon_{2}^{\mathrm{fT}}(\dot{\varepsilon })(\varepsilon_{2}-\varepsilon_{2}^{0T}(\dot{\varepsilon }))}{\varepsilon_{2}(\varepsilon_{2}^{\mathrm{fT}}(\dot{\varepsilon })-\varepsilon_{2}^{0T}(\dot{\varepsilon }))},\;d_{m}^{C}(\dot{\varepsilon })=\frac{\varepsilon_{\mathrm{mat}}^{\mathrm{fC}}(\dot{\varepsilon })(\varepsilon_{\mathrm{mat}}-\varepsilon_{\mathrm{mat}}^{0C})}{\varepsilon_{\mathrm{mat}}(\varepsilon_{\mathrm{mat}}^{\mathrm{fC}}(\dot{\varepsilon })-\varepsilon_{\mathrm{mat}}^{0C})} \end{array}$$

where the matrix dominated damage onset strains and ultimate failure strains are:

(36) $$\varepsilon_{2}^{0T}(\dot{\varepsilon })=\frac{Y^{T}({\dot{\varepsilon }}_{2})}{E_{2}},\;\varepsilon_{2}^{\mathrm{fT}}(\dot{\varepsilon })=\frac{2G_{2C}^{T}({\dot{\varepsilon }}_{2})}{Y^{T}({\dot{\varepsilon }}_{2})L^{c}},\;\varepsilon_{\mathrm{mat}}^{\mathrm{fC}}(\dot{\varepsilon })=\frac{2G_{\mathrm{matC}}^{C}({\dot{\varepsilon }}_{2})}{\sigma_{\mathrm{mat}}^{0C}L^{c}}$$

This damage model was implemented into Abaqus/Explicit through the VUMAT, and the element removal of the EHM was controlled with a state variable SDV34. The material properties of the EHM at the reference strain rate that were acquired by referring to Reference [62] and manufacturers are listed in Table 5.

#### 2.2.4. Interlayer Interface

Zero-thickness cohesive elements were adopted to simulate the interlayer interface. For the interlayer interface, mechanical behaviors in the normal direction, as well as the first and second tangential directions, were defined. Equation (37) was used to characterize the stress–strain relationship of the interlayer interface before damage:

(37) $$\left[\begin{array}{c} t_{n}\\ t_{s}\\ t_{t} \end{array}\right]=\left[\begin{array}{ccc} K_{n} & & \\ & K_{s} & \\ & & K_{t} \end{array}\right]\left[\begin{array}{c} \delta_{n}\\ \delta_{s}\\ \delta_{t} \end{array}\right]$$

where n, s, and t signify the normal direction, first tangential direction, and second tangential direction, respectively. *t*_i_ and *δ*_i_ are traction stresses and strains, respectively. *K*_i_ (i = n, s, t) is the elastic modulus. The strains in each direction read as follows [37]:

(38) $$\delta_{n}=\frac{u_{n}}{T_{0}},\delta_{s}=\frac{u_{s}}{T_{0}},\delta_{t}=\frac{u_{t}}{T_{0}}$$

In which, *u*_i_ is the displacement between the top and bottom faces of the cohesive element in each direction. $T_{0}$ is the constitutive thickness of the cohesive element, which equals one.

When the stress components of cohesive elements satisfied the quadratic nominal stress criterion shown by Equation (39), damage was initiated.

(39) $$F_{\mathrm{in}}={\left\{\frac{\langle t_{n}\rangle }{t_{n}^{0}}\right\}}^{2}+{\left\{\frac{t_{s}}{t_{s}^{0}}\right\}}^{2}+{\left\{\frac{t_{t}}{t_{t}^{0}}\right\}}^{2}=1$$

where $F_{\mathrm{in}}$ is the failure index and $t_{i}^{0}$ denotes the stress components at the damage initiation. After $F_{\mathrm{in}}$ exceeded one, the material stiffness degraded linearly on the basis of $d_{\mathrm{co}}$ (see Equation (40)). The stress components were calculated according to Equation (41).

(40) $$d_{\mathrm{co}}=\frac{u_{m}^{f}(u_{m}^{\max}-u_{m}^{0})}{u_{m}^{\max}(u_{m}^{f}-u_{m}^{0})}$$

(41) $$\begin{array}{l} t_{n}=\left\{\begin{array}{c} (1-d_{\mathrm{co}})\bar{t_{n}},\;\;\bar{t_{n}}\geq 0\\ \bar{t_{n}},\;\;\;\;\;\;\;\;\;\;\;\;\bar{t_{n}}<0 \end{array}\right.\\ t_{s}=(1-d_{\mathrm{co}})\bar{t_{s}}\\ t_{t}=(1-d_{\mathrm{co}})\bar{t_{t}} \end{array}$$

where $u_{m}=\sqrt{{\langle u_{n}\rangle }^{2}+u_{s}^{2}+u_{t}^{2}}$; *u*_i_ is the displacement component; $u_{m}^{0}$, $u_{m}^{f}$, and $u_{m}^{\max}$ represent the damage onset displacement, the ultimate failure displacement, and the maximum displacement in the calculation, respectively. ${\bar{t}}_{i}$ is the undamaged stress components.

When the absorbed energy met the power-law criterion (see Equation (42)), the cohesive element failed, and a crack was formed in the interface.

(42) $${\left(\frac{G_{n}}{G_{n}^{C}}\right)}^{\beta_{1}}+{\left(\frac{G_{s}}{G_{s}^{C}}\right)}^{\beta_{2}}+{\left(\frac{G_{t}}{G_{t}^{C}}\right)}^{\beta_{3}}=1$$

where *G*_n_, *G*_s_, and *G*_t_ are fracture toughness, and *G*_n_^C^, *G*_s_^C^, and *G*_t_^C^ denote the critical fracture toughness.$\beta_{1}$, $\beta_{2}$, and $\beta_{3}$ are material constants. The mechanical properties of the interlayer interface are summarized in Table 6 [15,16].

## 3. Drilling Experiments and Simulation Validations

In this study, drilling experiments were performed to validate the multiscale numerical model. The material removal process at the hole exit during the drilling of multidirectional CFRPs was experimentally observed, and the thrust force was measured. These experiment results were compared with the simulation outputs. Details of the experimental setups and simulation validations are described in this section.

### 3.1. Experimental Setups

**Figure 5:**
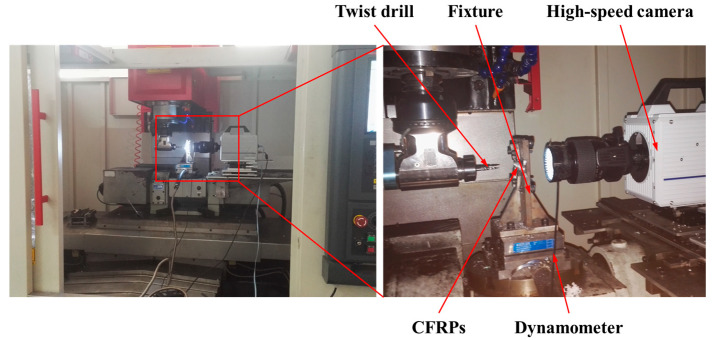
Details of the CFRP drilling experiment.

The drilling experiment was conducted on a GONA five-axis drilling center, featuring a maximum spindle speed of up to 8000 rpm. Figure 5 illustrates the specific experimental configurations. Throughout the experiments, the CFRPs were securely clamped to a dynamometer via a specially designed fixture, where the dynamometer was stabilized to ensure steady drilling processes. Considering that support at the hole exit impacts drilling quality, pre-drilled holes with the 14 mm diameter were processed in the fixture beneath the drilling area, minimizing any extraneous effects. A high-speed camera (PHOTRON SA5 (Photron, Tokyo, Japan)) captured the drilling activity at the hole exit, while a three-axis dynamometer (Kistler 9257B (Kistler, Winterthur, Switzerland)) measured the cutting forces. Additionally, a microscope (KEYENCE VH-Z50L (Keyence, Osaka, Japan)) was utilized to observe the burrs at the drilled exit of the workpiece.

The multidirectional CFRPs were manufactured by Shenyang Aircraft Corporation in China, and the layer configuration is [(−45/0/45/90)_2_/0/0]_s_, which is frequently applied in the aviation industry [4]. The thickness of the workpiece is 4 mm, and it contains 20 layers. The workpiece was cut into small sheets with dimensions of 150 mm × 20 mm × 4 mm to facilitate the clamping. Twist drills made of YG8 cemented carbide with diamond coating were utilized during the experiment. The drill bit geometries and drilling parameters were determined entirely based on the simulation. Additionally, the experiments with the same processing conditions were repeated to decrease the experimental errors.

**Figure 6:**
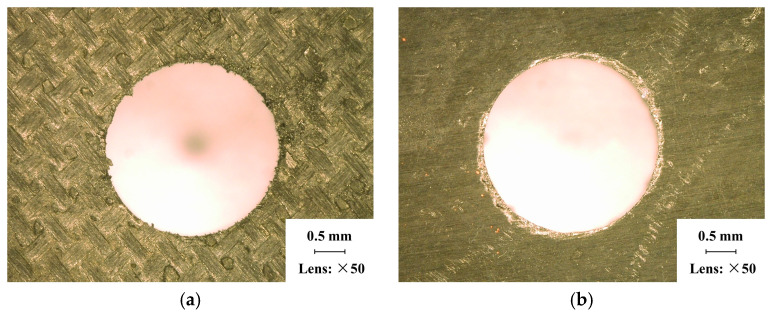
Details of the pre-drilled hole: (**a**) Hole entrance. (**b**) Hole exit.

Due to the fact that a pre-drilled hole was incorporated into the FE model on the workpiece, during the experiment, CFRPs were initially drilled using 3 mm diameter twist drills to process this hole. Subsequently, the pre-drilled hole was enlarged with 4 mm diameter twist drills to acquire results for simulating verification. Figure 6 depicts the drilling entrance and exit of the pre-drilled hole, revealing minimal damage and the absence of burrs. The pre-drilled hole processed with high quality ensured consistency between the initial states of the cutting area in the experiments and those in the FE model.

### 3.2. Removal Process of the Materials at Hole Exit During the CFRP Drilling

**Figure 7:**
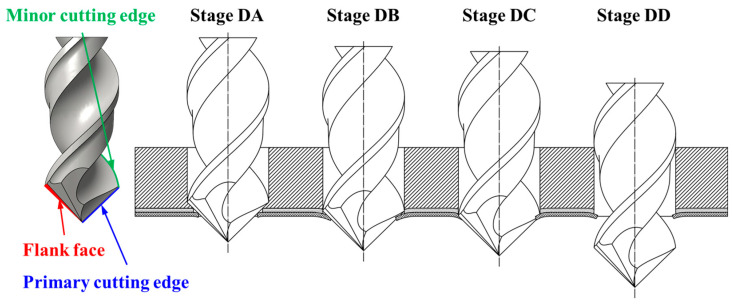
Illustration of the machining process at hole exit in the drilling of CFRPs with twist drill.

**Figure 8:**
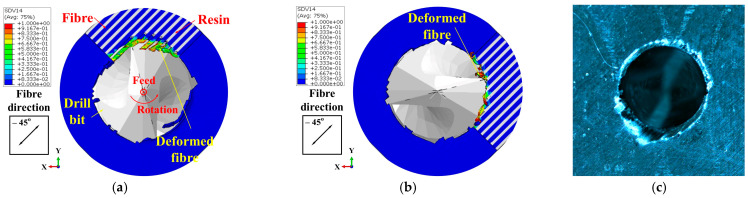
Cutting action of the drill bit on the materials at the drilling exit in Stage DA: (**a**) Results of Simulation-I. (**b**) Results of Simulation-II. (**c**) Experimental results.

**Figure 9:**
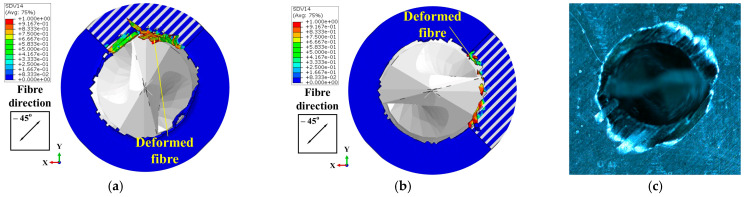
Cutting action of the drill bit on the materials at the drilling exit in Stage DB: (**a**) Results of Simulation-I. (**b**) Results of Simulation-II. (**c**) Experimental results.

**Figure 10:**
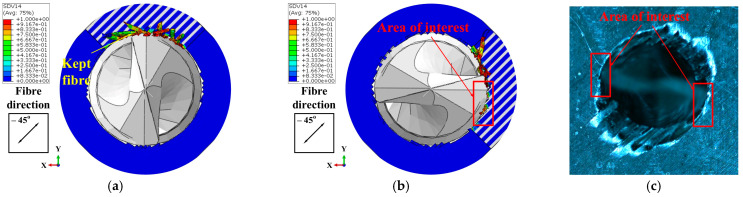
Cutting action of the drill bit on the materials at the drilling exit in Stage DC: (**a**) Results of Simulation-I. (**b**) Results of Simulation-II. (**c**) Experimental results.

**Figure 11:**
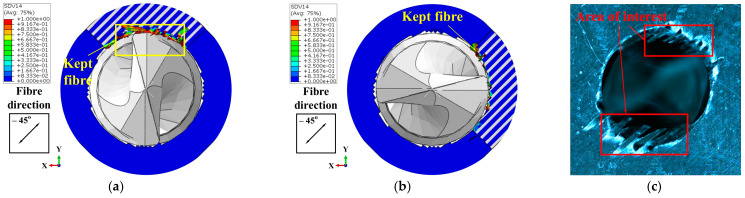
Cutting action of the drill bit on the materials at the drilling exit in Stage DD: (**a**) Results of Simulation-I. (**b**) Results of Simulation-II. (**c**) Experimental results.

In the FE model, the thickness of the workpiece was set to be 0.4 mm to enhance the computational efficiency, while the workpiece in the experiment was 4 mm thick. Therefore, the drilling process predicted by the FE model could not be directly compared with the experimental results. To address this issue, the predicted removal process of the materials at the hole exit was compared with the experimental observations to validate the FE model. Specifically, the drilling process at the hole exit was divided into four stages (see Figure 7) based on the actions of the drill bit cutting edges on the last ply of the CFRPs. Then, the predicted material removal processes in these four stages were compared with the experimental results individually to verify the numerical model. Stage DA marked the period when the primary cutting edges initiated the cutting of the last layer. During Stage DB, the majority of the primary cutting edges were involved in removing the final layer. Subsequently, in Stage DC, the minor cutting edges joined in the cutting process of the last layer. As the drill bit advanced, the hole was completed in Stage DD. During these four stages, the cutting actions of the drill bit on the materials at the drilling exit that were acquired by the numerical modeling and experimental observations are shown in Figure 8, Figure 9, Figure 10 and Figure 11. It should be noted that, in the simulation results, the blue and white areas in the fiber–resin combined zone symbolize the fibers and resins, respectively, and the rest zone of the workpiece shown in blue color represents the EHM. Moreover, the damage factor (SDV14) of the fiber is visualized to display the burrs.

The cutting action of the drill bit on the materials at the hole exit in Stage DA is illustrated in Figure 8. The simulation results have shown that some of the resins were removed during the cutting action of the primary cutting edge and the pressure applied by the flank face. The fibers were compelled to disperse and undergo deformation along the feed direction of the drill bit (i.e., out-of-plane deformation). Experimental findings indicated that the material was deformed and divided into strips due to the pressure and compression exerted by the drill bit, with portions of the material being removed.

Within the Stage DB shown in Figure 9, the cutting area surrounding the hole exit expanded with the drill bit feed. The simulation results revealed that nearly all the resins within this area were removed, while the fibers were well remained due to their out-of-plane deformation. Furthermore, as the drill bit rotated, the fibers in the drilling area bent in harmony with the rotational direction of the tool. The length of the kept fibers continued to increase. It was at this point that burrs began to form. For the experiments, the tearing of the material was propagated along the fiber direction under the action of the drill bit, which resulted in the rise of the length of the strip-like material. Meanwhile, similar to the simulation results, the material was bent in the direction of the tool rotation.

During Stage DC, the simulation outputs, as depicted in Figure 10a,b, demonstrated that the fibers within the highlighted area (i.e., the area with obtuse fiber cutting angles) were efficiently removed without generating burrs. Conversely, the remaining fibers persisted due to bending. The experimental outcomes shown in Figure 10c indicated that the material in the designated area was entirely removed, yielding a well-finished surface. However, a significant quantity of strip-like material was observed along the remaining circumference of the hole.

Regarding the drilling process in Stage DD, the simulation results in Figure 11a,b indicated that some of the fibers were removed, while the majority of the fibers in the marked area (i.e., the area with acute fiber cutting angles) remained well due to bending, ultimately contributing to the formation of the final burrs. For the experiments, the strip-like material formed in Stage DC was partially eliminated, with the remaining material transforming into burrs, thereby compromising the quality of the machined surface (see the marked area in Figure 11c).

From the aforementioned comparison, it can be deduced that the predicted drilling process at the hole exit aligns with the experimental findings. Moreover, the actions of the drill bit on the fibers and resins and the interactions between these two phases were successfully modeled by the developed FE model. The FE model effectively addresses the experimental limitation, as it is scarcely feasible to observe such damage formation for the mechanistic analysis.

In addition, this work defined the burr factor $k_{b}$ to assess the numerically predicted and experimentally obtained burrs quantitatively. The burr factors obtained by the simulation ($k_{b}^{s}$) and experiment ($k_{b}^{e}$) were:

(43) $$k_{b}^{s}=b\cdot \sum_{i=1}^{n}L_{i}/A_{\mathrm{HOL}}k_{b}^{e}=\sum_{i=1}^{n}A_{i}/A_{\mathrm{HOL}}$$

where *b* denotes the width of the burrs in the simulations; $\sum_{i=1}^{n}L_{i}$ is the length sum of the burrs acquired by simulations; and $A_{\mathrm{HOL}}$ represents the nominal area of the machined hole. $\sum_{i=1}^{n}A_{i}$ denotes the area of the burrs in the experimental results, and it is determined through the Autodesk CAD 2020 (Autodesk, San Francisco, CA, USA). Table 7 lists the experimental and simulation results of the burr factor at the hole exit. It is indicated that the prediction error is about 12.7%, which is small for a numerical investigation on CFRP cutting. The simulation outcomes are in acceptable agreement with the experimental outputs of this paper, and the predicted burr factor could also be verified by experimental results in the previous study [63].

### 3.3. The Thrust Force

To further validate the FE model, the drilling thrust forces acquired by simulation and experiment were compared. During the simulated drilling process, the length of the workpiece cut by the primary cutting edge reached its maximum in Stage DB. Therefore, the thrust force generated in this stage was named the maximum thrust force, and it was selected for the verification of the numerical model. The maximum thrust forces from the results of Simulation-I, Simulation-II, and the experiment are summarized in Table 7, which are about 8 N. Moreover, the prediction errors of the Simulation-I and -II are approximately 3.5% and 14%, respectively. These values are small enough to confirm that the simulated forces are well agreed with the experimental outputs. Therefore, the numerical model developed in this paper possesses the ability to accurately predict the burr distribution and thrust force during CFRP drilling.

## 4. Results and Discussion

Based on the proposed multiscale numerical model, the burr distribution during the drilling of multidirectional CFRPs at typical machining conditions was predicted, and the influences of drill bit geometries and drilling parameters on burrs were assessed.

### 4.1. Distribution of Burr During the Drilling of Multidirectional CFRPs

**Figure 12:**
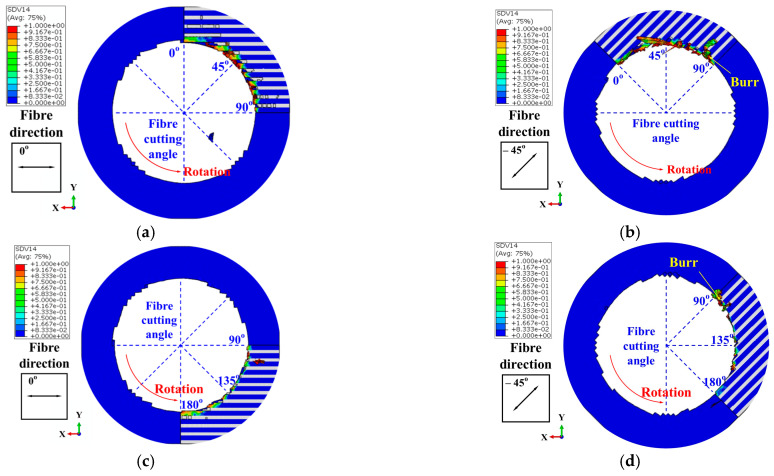
Burr distribution in the drilling of CFRPs with twist drill at 3000 rpm and 150 mm/min: (**a**) Results of the 0° layer in Simulation-I. (**b**) Results of the −45° layer in Simulation-I. (**c**) Results of the 0° layer in Simulation-II. (**d**) Results of the −45° layer in Simulation-II.

The simulated burr distribution in the drilling of CFRPs with a twist drill at 150 mm/min and 3000 rpm is illustrated in Figure 12. In which, the burrs produced at 0° to 90° fiber cutting angles were obtained from the results of the 0° layer and −45° layer in Simulation-I (see Figure 12a,b), while the results of the 0° layer and −45° layer in Simulation-II shown in Figure 12c,d represented the burrs induced at 90° to 180° fiber cutting angles. Since the 0° layer in the FE model is located above the −45° layer, the burrs produced in the 0° layer and −45° layer were utilized to predict the burrs induced at the hole wall and hole exit, respectively. In this context, the burr factors in the zones of 0° to 90° fiber cutting angles and 90° to 180° fiber cutting angles at the hole wall and hole exit in the drilling of multidirectional CFRPs were acquired, as listed in Table 8. Most notably, due to the fact that the FE model only simulated burrs on a quarter circle of the drilled hole, the values in Table 8 were obtained by doubling the results in Figure 12.

It can be found from the simulation results that the burrs were easily produced at hole exit, whereas there were virtually no burrs on the hole wall. The disparity in burr distribution between the hole exit and the hole wall can be attributed to variations in constraints. During the drilling of CFRPs, the material at the hole exit lacked support from below. Consequently, the fibers were susceptible to out-of-plane deformation under the pressure of the drill bit. Once deformation occurred, the fibers were difficult to remove, leading to the formation of burrs. In contrast, the fibers at the hole wall were supported by the material beneath, which mitigated out-of-plane deformation, resulting in the absence of burrs around the hole wall.

At the hole exit, the distribution of burrs depended on the fiber cutting angle. Specifically, the burrs occurred frequently at acute fiber cutting angles, whereas there were significantly fewer burrs under obtuse fiber cutting angles. When the fiber cutting angle was acute, the fibers were prone to bending radially outwards from the hole under the pressure of the cutting edges. Consequently, the cutting force on these fibers was decreased, leading to the production of burrs. Conversely, for obtuse fiber cutting angles, the fibers tended to bend radially inwards towards the hole. In this scenario, the stress in the fibers around the cutting area rapidly exceeded the ultimate strength of the fibers, resulting in a few burrs.

### 4.2. The Influences of Drill Bit Geometries and Drilling Parameters on the Burr

**Figure 13:**
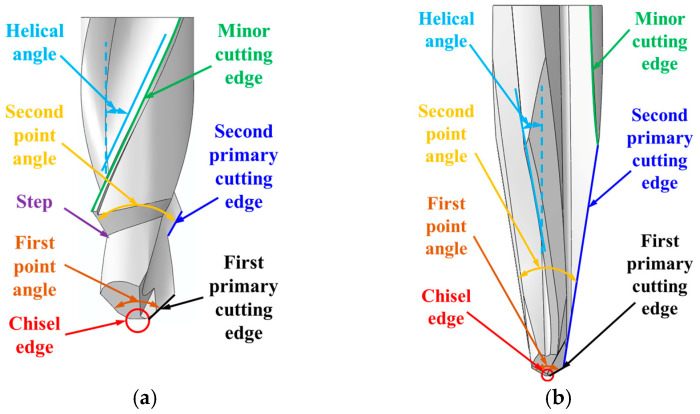
Geometric features of the drill bits: (**a**) Step drill. (**b**) One-shot drill.

**Figure 14:**
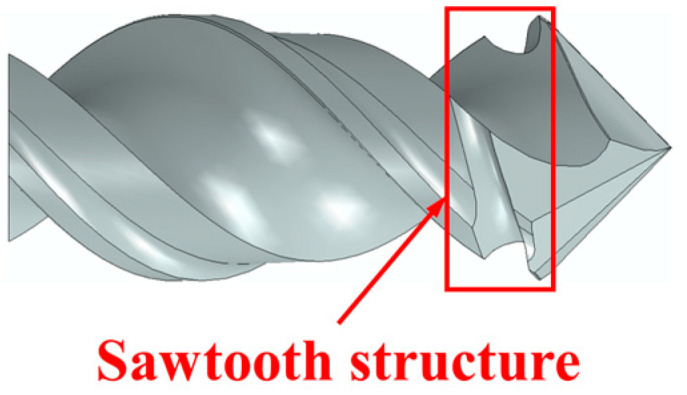
Illustration of the sawtooth structure of the twist drill with sawteeth.

In this section, typical drill bits, including twist drill, step drill, and one-shot drill, were selected to analyze the influences of tool geometries on the burr. The geometric features of the step drill and one-shot drill are shown in Figure 13, and their geometric parameters are listed in Table 9. Meanwhile, the investigation by Jia et al. [4] indicates that the burrs could be suppressed by setting the sawtooth structure (see Figure 14) between the primary cutting edge and the minor cutting edge of the drill bit. Therefore, twist drill with sawteeth, step drill with sawteeth, and one-shot drill with sawteeth were also involved in the parametric study. In addition, the burr distributions at various feed rates and spindle speeds were assessed. The determined tool structures and drilling parameters for the parametric study of the burr are summarized in Table 10. Moreover, the burrs were prone to being induced in the zones of 0° to 90° fiber cutting angles at the hole exit; therefore, the following studies were focused on the burrs produced in this area.

#### 4.2.1. The Effect of Tool Geometry

**Figure 15:**
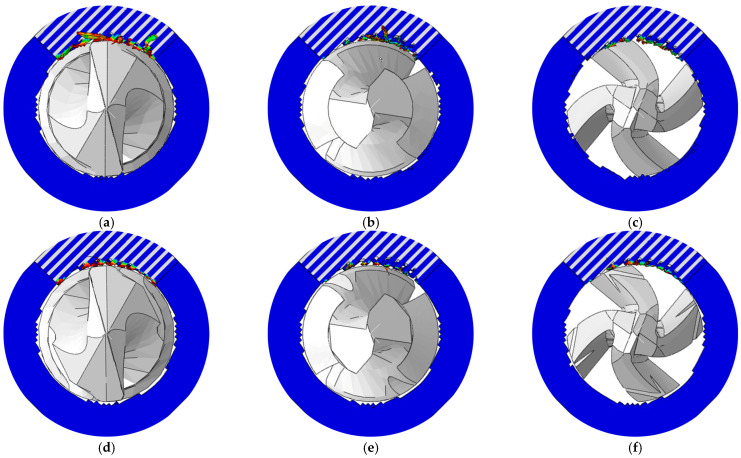
Burr distributions in the drilling of CFRPs at various tool geometries: (**a**) Twist drill; (**b**) step drill; (**c**) one-shot drill; (**d**) twist drill with sawteeth; (**e**) step drill with sawteeth; and (**f**) one-shot drill with sawteeth.

**Figure 16:**
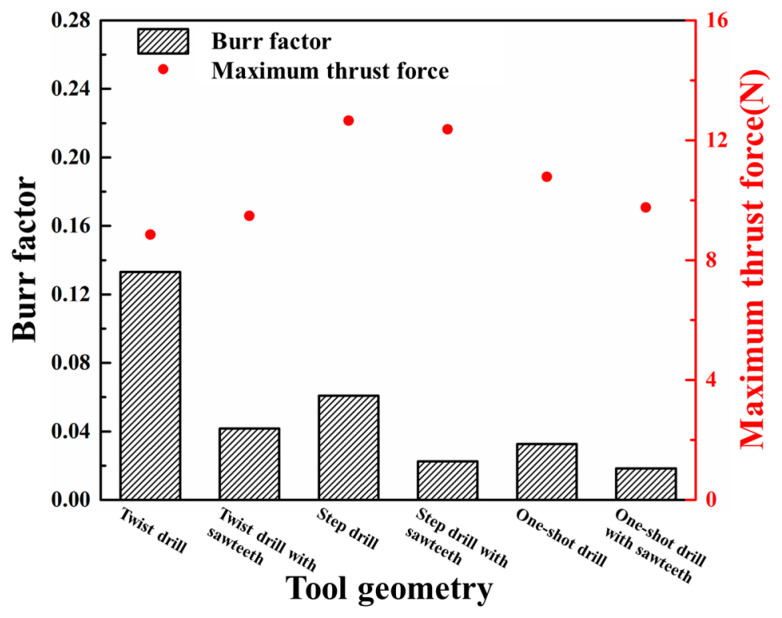
Variations in the burr factor and maximum thrust force with tool geometry.

The simulated burr distributions in the drilling of CFRPs using different drill bits at 3000 rpm and 150 mm/min are displayed in Figure 15. The variations in the burr factor and maximum thrust force with the tool geometry are shown in Figure 16. The results indicated that the sawtooth structure had less effect on the maximum thrust force. The reason could be attributed to the fact that the sawtooth structure was set between the primary cutting edge and the minor cutting edge, and the sawtooth structure had not participated in the drilling when the maximum thrust force was reached. The smallest and largest values of the maximum thrust force were obtained with the twist drill and step drill, respectively. Moreover, the change in the maximum thrust force with the tool geometry was not obvious, and it ranged from 8 N to 12 N. The difference between the maximum thrust forces induced by these drill bits is related to the variations in the point angle and helical angle of the tools.

**Figure 17:**
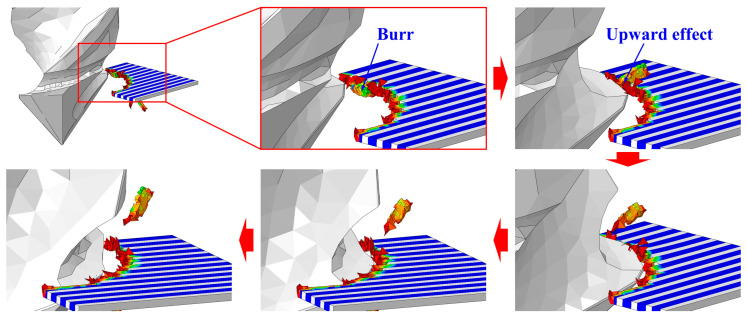
Removal process of burrs by drill bit with sawteeth structure (twist drill with sawteeth, 150 mm/min, and 3000 rpm).

For the drill bits without a sawtooth structure, the most serious burr damage was produced by the twist drill, and the burr factor was approximately 0.14. The burrs were suppressed when utilizing the step drill and the one-shot drill, and the burr factors were about 0.06 and 0.04. The minor flank of the step drill was larger than that of the twist drill, which increased the compression effect of the cutting edges on the workpiece, leading to reduced burrs around the processed hole. The second point angle of the one-shot drill was 20°, which was far less than that of the step drill and the point angle of the twist drill. With smaller point angles, the pushing effect of the tool on the fibers at the hole exit was weaker, and the fibers were less likely to undergo out-of-plane deformation, resulting in significantly decreased burrs. In addition, it could be concluded from Figure 16 that the burrs were suppressed after using tools with a sawtooth structure. The reason can be explained by analyzing the drilling process of the twist drill with a sawteeth frame by frame, as shown in Figure 17. After the initial burrs were formed in the drilling process, the sawtooth structure could exert an upward cutting effect on burrs during the downward feed of the tool. Due to the strong constraint effect of the material above the fibers at the hole exit, the fibers were less likely to undergo out-of-plane deformation, and they were easily removed; thereby, the burrs were inhibited. Since the twist drill has been commonly applied in industrial production while the burrs induced by it were the most serious, the following parametric studies were conducted with the twist drill.

#### 4.2.2. The Effect of Feed Rate

**Figure 18:**
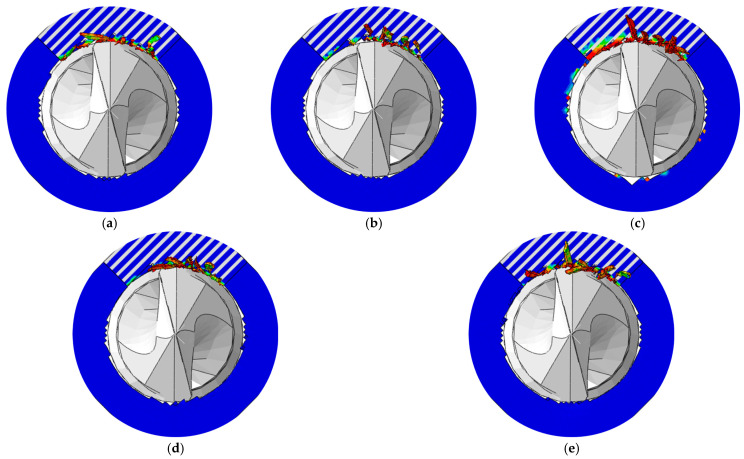
Burr distributions in the drilling of CFRPs at various feed rates: (**a**) 150 mm/min; (**b**) 250 mm/min; (**c**) 350 mm/min; (**d**) 450 mm/min; and (**e**) 550 mm/min.

**Figure 19:**
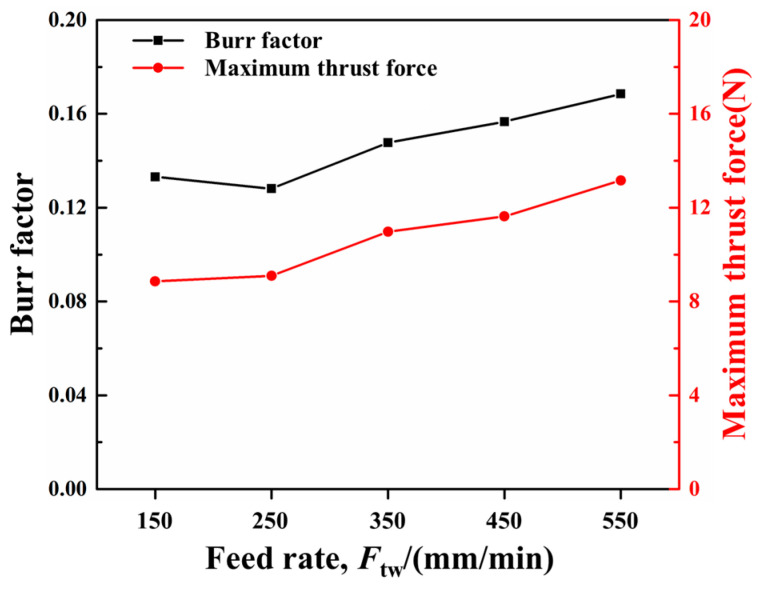
Variations in the burr factor and maximum thrust force with feed rate.

Figure 18 illustrates the predicted burr distributions in drilling CFRPs with the twist drill at various feed rates and 3000 rpm spindle speed. The changes in the burr factor and maximum thrust force with the feed rate are shown in Figure 19. It can be seen from the results that the burr factor raised from about 0.14 to about 0.16 with the increase in the feed rate from 150 mm/min to 550 mm/min, and the maximum thrust force gradually increased from around 8 N to about 13 N. With higher feed rates, the pushing effect of the tool on the fibers was enhanced, leading to great out-of-plane deformation of the fibers. The fibers were hence more difficult to remove, and the burrs were increased. In addition, the increase in feed rate raised the cutting amount per tooth of the tool, thereby resulting in large maximum thrust forces.

#### 4.2.3. The Effect of Spindle Speed

**Figure 20:**
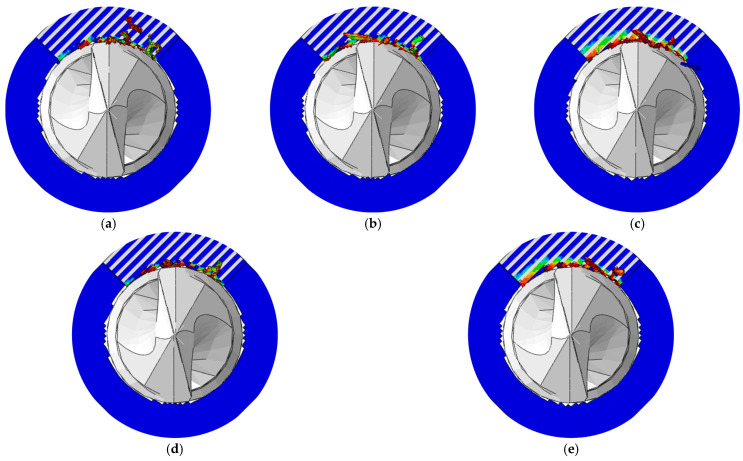
Burr distributions in the drilling of CFRPs at various spindle speeds: (**a**) 2000 rpm; (**b**) 3000 rpm; (**c**) 4000 rpm; (**d**) 5000 rpm; and (**e**) 6000 rpm.

**Figure 21:**
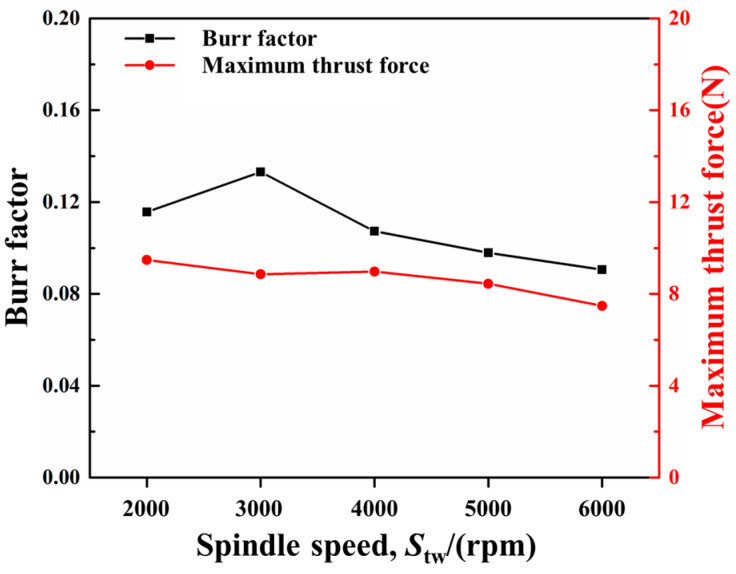
Variations in the burr factor and maximum thrust force with spindle speed.

The predicted burr distributions in drilling CFRPs using the twist drill at a 150 mm/min feed rate and various spindle speeds are shown in Figure 20. The variations in the burr factor and maximum thrust force with the spindle speed are illustrated in Figure 21. With the increase in the spindle speed from 2000 rpm to 6000 rpm, the burr factor decreased from around 0.14 to around 0.09, and the maximum thrust force progressively reduced from around 10 N to about 8 N. The rise of the spindle speed made the cutting times of the tool on the fibers greater, which led to a faster accumulation of stress in the fibers. In this case, the fiberr inner stress was more likely to exceed its ultimate strength, and the fibers could be easily removed, resulting in fewer burrs. Moreover, the cutting amount per tooth of the tool was decreased at higher spindle speeds; thus, the maximum thrust force was reduced.

## 5. Conclusions

A 3D numerical model with multiscale modeling was developed in this paper to research the distributions of burrs during the CFRP drilling at various processing conditions. Using this numerical model, the material removal process of the fibers and resins at the hole exit and the thrust force were predicted, and the simulation results agree well with the experimental outputs. Then, the burr distributions under different tool structures and processing parameters were assessed. The main conclusions are as follows:

1. The proposed FE model could accurately predict the cutting actions of the drill bit on the fibers and resins during the CFRP drilling, as well as the formation process of the burrs at the drilling exit. The prediction error of the burr factor at the drilling exit is about 12.7%. The simulated thrust forces are well agreed with the experimental thrust forces, and the prediction errors are not greater than 14%.
2. The simulation results indicate that the burrs were easily produced at hole exit, whereas there were virtually no burrs on the hole wall. At the hole exit, the burrs occurred frequently at acute fiber cutting angles, whereas there were significantly fewer burrs under obtuse fiber cutting angles.
3. The most serious burr damage is produced by the twist drill, and the burr factor in the zones of 0° to 90° fiber cutting angles at the hole exit is approximately 0.14. The burrs could be suppressed by utilizing the step drill and the one-shot drill. The sawtooth structure could exert an upward cutting effect on burrs during the downward feed of the tool; thus, it is helpful for the inhibition of burrs. For the CFRP drilling with twist drills, higher feed rates would lead to increased out-of-plane deformation of the fibers, and more burrs are produced. The rise of the spindle speed would result in a faster accumulation of stress in the fibers, and the fiber inner stress is more likely to exceed its ultimate strength, leading to fewer burrs.

## Figures and Tables

**Table 1:** Details of the twist drill and drilling parameters.

Drill Bit			Drilling Parameters	
Diameter	Point Angle	Helical Angle	Spindle Speed, *S*_tw_	Feed Rate, *F*_tw_
4 mm	90°	34°	3000 rpm	150 mm/min

**Table 2:** Mechanical properties of carbon fiber [35,36].

Properties	Value	Properties	Value	Properties	Value
*E*_f1_ (GPa)	294	$X_{f0}^{T}$ (MPa)	5880	$G_{f1C}^{T}$ (kJ/m^2^)	60
*E*_f2_ = *E*_f3_ (GPa)	15	$X_{f0}^{C}$ (MPa)	3850	$G_{f1C}^{C}$ (kJ/m^2^)	40
*G*_f12_ = *G*_f13_ (GPa)	32	$Y_{f0}^{T}=Z_{f0}^{T}$ (MPa)	200	$G_{f2C}^{T}=G_{f3C}^{T}$ (kJ/m^2^)	2
*G*_f23_ (GPa)	5.9	$Y_{f0}^{C}=Z_{f0}^{C}$ (MPa)	600	$G_{f2C}^{C}=G_{f3C}^{C}$ (kJ/m^2^)	6
*ν*_f12_ = *ν*_f13_	0.25	*S*_f12_*= S*_f13_ (MPa)	140	*ρ* (g/cm^3^)	1.8
*ν*_f23_	0.28	*S*_f23_ (MPa)	120		

**Table 3:** Mechanical properties of resin at reference strain rate [46].

Properties	Value	Properties	Value	Properties	Value	Properties	Value
*E*_m_ (GPa)	4.2	*σ*_me_ (MPa)	70	${\dot{\varepsilon }}_{0}$ (s^−1^)	10^−4^	*ρ* (g/cm^3^)	0.98
*ν*_m_	0.4	${\bar{\varepsilon }}_{0}^{\mathrm{pl}}$	0.02	*G*_mC_ (kJ/m^2^)	1		

**Table 4:** Strain-rate-dependent damage initiation criteria for the EHM.

Failure Mode	Formula
Fiber tensile failure (*σ*_1_ ≥ 0)	$F_{f}^{T}={\left(\frac{\sigma_{1}}{X^{T}}\right)}^{2}+{\left(\frac{\tau_{12}}{S_{12}({\dot{\varepsilon }}_{12})}\right)}^{2}+{\left(\frac{\tau_{13}}{S_{13}({\dot{\varepsilon }}_{13})}\right)}^{2}=1$
Fiber compressive failure (*σ*_1_ < 0)	$F_{f}^{C}={\left(\frac{\sigma_{1}}{X^{C}}\right)}^{2}+{\left(\frac{\tau_{12}}{S_{12}({\dot{\varepsilon }}_{12})}\right)}^{2}+{\left(\frac{\tau_{13}}{S_{13}({\dot{\varepsilon }}_{13})}\right)}^{2}=1$
Matrix tensile failure (*σ*_2_ ≥ 0)	$F_{m}^{T}={\left(\frac{\sigma_{2}}{Y^{T}({\dot{\varepsilon }}_{2})}\right)}^{2}+{\left(\frac{\tau_{12}}{S_{12}({\dot{\varepsilon }}_{12})}\right)}^{2}+{\left(\frac{\tau_{23}}{S_{23}({\dot{\varepsilon }}_{23})}\right)}^{2}=1$
Matrix compressive failure (*σ*_2_ < 0)	$F_{m}^{C}={\left(\frac{\tau_{t}}{S_{23}^{A}(\dot{\varepsilon })-\mu_{t}\sigma_{n}}\right)}^{2}+{\left(\frac{\tau_{s}}{S_{12}({\dot{\varepsilon }}_{12})-\mu_{s}(\dot{\varepsilon })\sigma_{n}}\right)}^{2}=1$

**Table 5:** Quasi-static mechanical properties of the EHM [62].

Properties	Value	Properties	Value	Properties	Value
*E*_1_ (GPa)	178	*X*^T^ (MPa)	2980	$G_{1C}^{T}$ (kJ/m^2^)	91.6
*E*_2_ = *E*_3_ (GPa)	9.5	*X*^C^ (MPa)	1450	$G_{1C}^{C}$ (kJ/m^2^)	79.9
*G*_12_ = *G*_13_ (GPa)	6.33	*Y*^T^ (MPa)	110	$G_{2C}^{T}$ (kJ/m^2^)	0.22
*G*_23_ (GPa)	4.21	*Y*^C^ (MPa)	350	$G_{\mathrm{matC}}^{C}$ (kJ/m^2^)	1.1
*ν*_12_ = *ν*_13_	0.29	*S*_12_*= S*_13_ (MPa)	90	*ρ* (g/cm3)	1.53
*ν*_23_	0.37	*S*_23_ (MPa)	40	*θ* (°)	53

**Table 6:** Mechanical properties of the interlayer interface [15,16].

Properties	Value	Properties	Value
$K_{n}$ (N/mm^3^)	1 × 10^6^	$G_{n}^{C}$ (N/mm)	0.33
$K_{s}=K_{t}$ (N/mm^3^)	1 × 10^6^	$G_{s}^{C}=G_{t}^{C}$ (N/mm)	1.2
$t_{n}^{0}$ (MPa)	60	*ρ* (g/cm^3^)	1.53
$t_{s}^{0}=t_{t}^{0}$ (MPa)	110	$\beta_{1}$ = $\beta_{2}$ = $\beta_{3}$	1.6

**Table 7:** Experimental and simulation results of the burr factor at the hole exit and the maximum thrust force in the CFRP drilling.

	Experiment	Simulation	Error
Burr factor at the hole exit	0.158	0.178	12.7%
Maximum thrust force	8.6 N	8.9 N (Simulation-I)	3.5% (Simulation-I)
		7.4 N (Simulation-II)	14.0% (Simulation-II)

**Table 8:** Predicted burr factors at different zones (twist drill, 150 mm/min, and 3000 rpm).

	Hole Wall	Hole Exit
0° to 90° fiber cutting angle (Simulation-I)	0.046	0.133
90° to 180° fiber cutting angle (Simulation-II)	0.003	0.045

**Table 9:** Geometric parameters of the step drill and one-shot drill.

Parameter		Value
Step drill	Diameter (mm)	4
	Diameter of step (mm)	2.5
	First point angle (°)	90
	Second point angle (°)	90
	Helical angle (°)	20
One-shot drill	Diameter (mm)	4
	First point angle (°)	118
	Second point angle (°)	20
	Helical angle (°)	6

**Table 10:** Details of the tool structures and drilling parameters in the parametric study.

Parameter	Setup
Drill bit	twist drill, twist drill with sawteeth, step drill, step drill with sawteeth, one-shot drill, and one-shot drill with sawteeth
Feed rate, *F*_tw_/(mm/min)	150, 250, 350, 450, and 550
Spindle speed, *S*_tw_/(rpm)	2000, 3000, 4000, 5000, and 6000

## Data Availability

The original contributions presented in this study are included in the article. Further inquiries can be directed to the corresponding author.
